# Bis[hexa­aquacobalt(II)] 25,26,27,28-tetra­hydr­oxy-2,8,14,19-tetra­thia­calix[4]arene-5,11,17,23-tetra­sulfonate monohydrate

**DOI:** 10.1107/S1600536808011781

**Published:** 2008-05-10

**Authors:** Haruo Akashi, Misato Ichikawa

**Affiliations:** a Research Institute of Natural Sciences, Okayama University of Science, Ridai-cho, Okayama 700-0005, Japan

## Abstract

In the crystal structure of the title compound, [Co(H_2_O)_6_]_2_(C_24_H_12_O_16_S_8_)·H_2_O, the thia­calix[4]arenetetra­sulfonate (= TCAS^4−^) anions adopt a cone-type conformation with an additional water mol­ecule as a guest mol­ecule in the hydro­phobic cavity. The TCAS^4−^ anions are arranged in layers in an up–down fashion. These anionic layers alternate with cationic layers consisting of rather regular octahedral cations (symmetry *m*). Several medium O—H⋯O hydrogen-bond inter­actions exist between the aqua ligands of the [Co(H_2_O)_6_]^2+^ cations and the O atoms of the sulfonate groups. In addition to the two crystallographically different Co atoms, two S and four O atoms are situated on mirror planes.

## Related literature

For the structure of sodium thia­calix[4]arene tetra­sulfonate monohydrate, see: Akashi & Yamauchi (2003 ▶), and for the Cd salt of the same anion, see: Zhao *et al.* (2005 ▶). Assemblies of thia­calix[4]arene tetra­sulfonates with several transition metal ions were described by Guo *et al.* (2004 ▶).
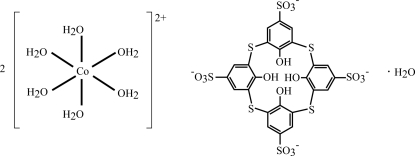


## Experimental

### 

#### Crystal data

[Co(H_2_O)_6_]_2_(C_24_H_12_O_16_S_8_)·H_2_O
*M* *_r_* = 1164.89
Monoclinic, 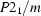

*a* = 11.9955 (5) Å
*b* = 14.0628 (10) Å
*c* = 12.8907 (11) Å
β = 95.638 (3)°
*V* = 2164.0 (3) Å^3^
*Z* = 2
Mo *K*α radiation
μ = 1.25 mm^−1^
*T* = 93 K
0.40 × 0.40 × 0.10 mm

#### Data collection

Rigaku R-AXIS-IV diffractometer
Absorption correction: multi-scan (*ABSCOR*; Higashi, 1995 ▶) *T*_min_ = 0.767, *T*_max_ = 0.883
15194 measured reflections
4319 independent reflections
3640 reflections with *F* ^2^ > 2σ(*F* ^2^)
*R*_int_ = 0.047

#### Refinement

*R*[*F* ^2^ > 2σ(*F* ^2^)] = 0.044
*wR*(*F* ^2^) = 0.144
*S* = 1.00
4296 reflections
320 parameters
H-atom parameters constrained
Δρ_max_ = 1.65 e Å^−3^
Δρ_min_ = −0.81 e Å^−3^

### 

Data collection: *PROCESS-AUTO* (Rigaku, 1998 ▶); cell refinement: *PROCESS-AUTO*; data reduction: *CrystalStructure* (Rigaku/MSC, 2006 ▶); program(s) used to solve structure: *SHELXS97* (Sheldrick, 2008 ▶); program(s) used to refine structure: *CrystalStructure*; molecular graphics: *ORTEP-3 for Windows* (Farrugia, 1997 ▶); software used to prepare material for publication: *CrystalStructure*.

## Supplementary Material

Crystal structure: contains datablocks global, I. DOI: 10.1107/S1600536808011781/wm2177sup1.cif

Structure factors: contains datablocks I. DOI: 10.1107/S1600536808011781/wm2177Isup2.hkl

Additional supplementary materials:  crystallographic information; 3D view; checkCIF report

## Figures and Tables

**Table 1:** Selected bond lengths (Å)

Co1—O9	2.079 (3)
Co1—O10	2.064 (3)
Co1—O11	2.085 (2)
Co1—O12	2.088 (2)
Co2—O13	2.076 (3)
Co2—O14	2.090 (3)
Co2—O15	2.078 (2)
Co2—O16	2.085 (2)

**Table 2:** Hydrogen-bond geometry (Å, °)

*D*—H⋯*A*	*D*—H	H⋯*A*	*D*⋯*A*	*D*—H⋯*A*
O1—H5⋯O1^i^	0.78	2.17	2.877 (3)	151
O2—H6⋯O1	0.88	2.26	3.082 (3)	156
O2—H6⋯O12^ii^	0.88	2.71	3.154 (3)	113
O9—H7⋯O4^iii^	0.82	2.06	2.878 (3)	173
O9—H7⋯O4^iii^	0.82	2.06	2.878 (3)	173
O10—H8⋯O8^iv^	0.93	1.83	2.729 (2)	163
O10—H8⋯O8^iv^	0.93	1.83	2.729 (2)	163
O11—H9⋯O3^v^	0.81	1.95	2.741 (3)	165
O11—H10⋯O7^v^	0.77	2.04	2.795 (3)	169
O12—H11⋯O5	0.91	1.86	2.764 (3)	173
O12—H12⋯O6^iv^	0.82	2.00	2.812 (3)	174
O13—H13⋯O7^vi^	0.81	1.95	2.739 (2)	165
O13—H13⋯O7^vi^	0.81	1.95	2.739 (2)	165
O14—H14⋯O5	0.78	2.05	2.818 (2)	172
O14—H14⋯O5	0.78	2.05	2.818 (2)	172
O15—H15⋯O6^vii^	0.84	1.91	2.739 (3)	170
O15—H16⋯O4^v^	0.87	1.89	2.743 (3)	164
O16—H17⋯O8^viii^	0.77	2.01	2.764 (3)	166
O16—H18⋯O3^viii^	0.84	1.92	2.719 (3)	159

Symmetry codes: (i) 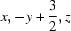; (ii) 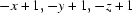; (iii) 
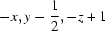
; (iv) 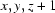; (v) 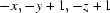; (vi) 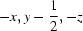; (vii) 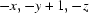; (viii) 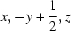.

**Fig. 1.**
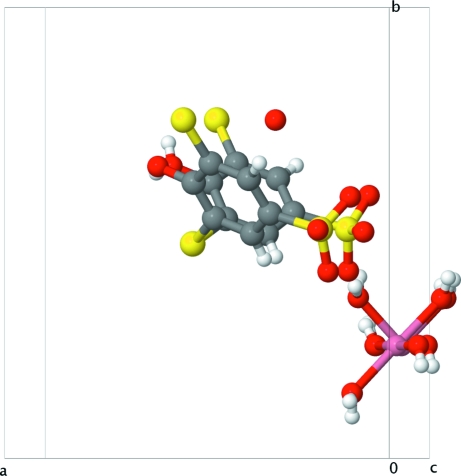
The molecular entities of (I) with the atom-labelling scheme and displacement ellipsoids drawn at 50% probability level. Labelling of H atoms has been omitted for clarity. [Symmetry code: (i) x, 1/2 - y, z.]

**Fig. 2.**
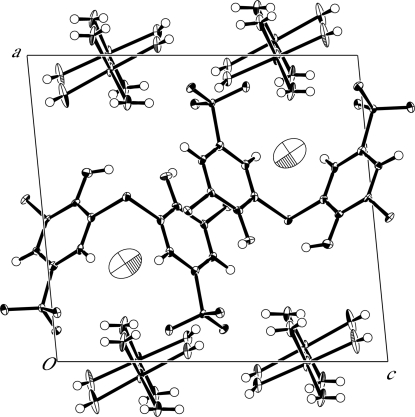
The projection of the structure of (I) along the b axis.

## supplementary crystallographic information

### Comment

The water soluble thiacalix[4]arene tetrasulfonate anion (= TCAS^4-^) is well-known as a second-sphere ligand of various transition metal coordination compounds containing complex metal aqua cations (Zhao, 2005). In the title compound, [Co(H_2_O)_6_]_2_(S_8_C_24_O_16_H_12_)^.^H_2_O, (I), the second-sphere TCAS^4-^ anionic ligands are linked to the aqua ligands of [Co(H_2_O)_6_] via several medium O-H···H hydrogen bonding interactions (see Table 2).

In the crystal structure of (I) the TCAS^4-^ molecules adopt the cone-type conformation. The Co^2+^ ions are not directly bonded to the TCAS^4-^ moieties, but exist as two octahedral aqua complexes, [Co(H_2_O)_6_]^2+^, both with *m* symmetry (Figure 1, Table 1). Layers of TCAS^4-^ anions are arranged in an up-down fashion and alternate with cationic layers that contain the [Co(H_2_O)_6_]^2+^ cations (Figure 2).

The TCAS^4-^ molecule include a water molecule as a guest-molecule in the hydrophobic cavity. Interactions between the H atoms of the aqua ligands of [Co(H_2_O)_6_]^2+^ and of O atoms of the TCAS^4-^ moieties constitute a three-dimensional hydrogen bond network, and from there the second-sphere coordination is established.

### Experimental

A solution of Na_4_(S_8_C_24_O_16_H_12_)^.^14H_2_O (=Na_4_TCAS^.^14H_2_O) in 2 *M* HCl and a 2*M* HCl solution containing CoCl_2_^.^6H_2_O were mixed in the molar ratio (Co/Na_4_TCAS = 5), poured into a vial and stirred for 1 h. This solution was then kept at room temperature from which pale pink crystals of (I) were obtained within a few days. Elemental analysis of (I) is in agreement with the refined structure model. Found: C 24.85, H 2.89%; calculated for (I): C 24.74, H 3.29%.

### Refinement

Refinement was performed using 4296 reflections (5 < 2θ < 51°) out of 4319 independent reflections. The positions of all the H atoms were initially located from a  difference map. All H atoms were refined by using the riding model approximation with C—H distances in the range 0.77–0.93Å. The isotropic displacement parameters for all H atoms were fixed at 1.2 times the value of the equivalent isotropic displacement parameter of their carrier atom. The H atoms attached to the water molecule in the hydrophobic cavity of thiacalix[4]arenetetrasulfonate could not be found from a difference map. Therefore the positions of the H atoms were not refined. The maximum of the remaining electron density is located 2.66Å away from atom O15.

### Crystal data

**Table:**

[Co(H_2_O)_6_]_2_(C_24_H_12_O_16_S_8_)·H_2_O	*F*_000_ = 1192.00
*M**_r_* = 1164.89	*D*_x_ = 1.788 Mg m^−^^3^
Monoclinic, *P*2_1_/*m*	Mo *K*α radiation λ = 0.71069 Å
Hall symbol: -P 2yb	Cell parameters from 14562 reflections
*a* = 11.9955 (5) Å	θ = 2.2–27.5º
*b* = 14.0628 (10) Å	µ = 1.25 mm^−^^1^
*c* = 12.8907 (11) Å	*T* = 93 K
β = 95.638 (3)º	Block, pale pink
*V* = 2164.0 (3) Å^3^	0.40 × 0.40 × 0.10 mm
*Z* = 2	

### Data collection

**Table:**

Rigaku R-AXIS-IV diffractometer	3640 reflections with *F*^2^ > 2σ(*F*^2^)
Detector resolution: 10.00 pixels mm^-1^	*R*_int_ = 0.047
ω scans	θ_max_ = 25.5º
Absorption correction: multi-scan(ABSCOR; Higashi, 1995)	*h* = −15→15
*T*_min_ = 0.767, *T*_max_ = 0.883	*k* = −17→17
15194 measured reflections	*l* = −16→16
4319 independent reflections	

### Refinement

**Table:**

Refinement on *F*^2^	H-atom parameters constrained
*R*[*F*^2^ > 2σ(*F*^2^)] = 0.044	*w* = 1/[0.0031*F*_o_^2^ + σ(*F*_o_^2^)]/(4*F*_o_^2^)
*wR*(*F*^2^) = 0.144	(Δ/σ)_max_ < 0.001
*S* = 1.00	Δρ_max_ = 1.65 e Å^−^^3^
4296 reflections	Δρ_min_ = −0.81 e Å^−^^3^
320 parameters	Extinction correction: none

### Special details

**Table:**

Refinement. Refinement was performed using 4296 reflections (5 < 2θ < 51°). The weighted *R*-factor (*wR*) and goodness of fit (*S*) are based on *F*^2^. *R* factor (gt) are based on *F*. The threshold expression of *F*^2^ > 2σ(*F*^2^) is used only for calculating *R* factor (gt).

### Fractional atomic coordinates and isotropic or equivalent isotropic displacement parameters (Å^2^)

**Table:**

	*x*	*y*	*z*	*U*_iso_*/*U*_eq_	
Co1	0.04693 (6)	0.2500	0.75294 (5)	0.01428 (17)	
Co2	0.00632 (6)	0.2500	0.24647 (5)	0.01639 (17)	
S1	0.50722 (9)	0.7500	0.56024 (9)	0.0120 (2)	
S2	0.53799 (7)	0.47539 (6)	0.25884 (6)	0.01238 (19)	
S3	0.52961 (10)	0.7500	−0.04805 (9)	0.0129 (2)	
S4	0.15582 (6)	0.50788 (5)	0.45489 (6)	0.00843 (18)	
S5	0.17521 (6)	0.50752 (5)	−0.03055 (6)	0.00836 (18)	
O1	0.60482 (18)	0.64771 (18)	0.38463 (19)	0.0174 (5)	
O2	0.61612 (19)	0.64728 (17)	0.14682 (18)	0.0162 (5)	
O3	0.10300 (19)	0.50174 (16)	0.34755 (17)	0.0122 (5)	
O4	0.10609 (19)	0.58262 (17)	0.51376 (17)	0.0130 (5)	
O5	0.15942 (19)	0.41514 (16)	0.50631 (17)	0.0135 (5)	
O6	0.1697 (2)	0.50641 (16)	−0.14431 (18)	0.0132 (5)	
O7	0.10106 (19)	0.57942 (16)	0.00710 (17)	0.0121 (5)	
O8	0.15982 (19)	0.41379 (16)	0.01357 (17)	0.0117 (5)	
O9	−0.0351 (3)	0.250000 (10)	0.6031 (2)	0.0206 (9)	
O10	0.1310 (4)	0.250000 (10)	0.9006 (2)	0.0393 (12)	
O11	−0.0636 (2)	0.35446 (18)	0.79486 (19)	0.0257 (6)	
O12	0.1547 (2)	0.35660 (17)	0.71050 (18)	0.0184 (6)	
O13	−0.0660 (4)	0.250000 (10)	0.0934 (2)	0.0380 (11)	
O14	0.0769 (3)	0.250000 (10)	0.4013 (2)	0.0178 (8)	
O15	−0.1064 (2)	0.35324 (17)	0.28526 (18)	0.0201 (6)	
O16	0.1180 (2)	0.14395 (18)	0.21098 (19)	0.0240 (6)	
O17	0.3199 (9)	0.7500	0.2349 (9)	0.154 (4)	
C1	0.5066 (2)	0.6116 (2)	0.4082 (2)	0.0123 (7)	
C2	0.4618 (2)	0.5311 (2)	0.3545 (2)	0.0107 (7)	
C3	0.3575 (2)	0.4978 (2)	0.3728 (2)	0.0113 (7)	
C4	0.2960 (2)	0.5438 (2)	0.4440 (2)	0.0103 (7)	
C5	0.3419 (2)	0.6201 (2)	0.5015 (2)	0.0107 (7)	
C6	0.4478 (2)	0.6531 (2)	0.4843 (2)	0.0102 (7)	
C7	0.5189 (2)	0.6115 (2)	0.1034 (2)	0.0114 (7)	
C8	0.4647 (2)	0.6537 (2)	0.0126 (2)	0.0098 (7)	
C9	0.3610 (2)	0.6200 (2)	−0.0297 (2)	0.0093 (7)	
C10	0.3118 (2)	0.5430 (2)	0.0163 (2)	0.0106 (7)	
C11	0.3666 (2)	0.4982 (2)	0.1027 (2)	0.0108 (7)	
C12	0.4701 (2)	0.5325 (2)	0.1461 (2)	0.0105 (7)	
H1	0.3306	0.4470	0.3377	0.013*	
H2	0.2996	0.6492	0.5510	0.013*	
H3	0.3269	0.6542	−0.0940	0.011*	
H4	0.3380	0.4414	0.1315	0.013*	
H5	0.6164	0.6994	0.4048	0.021*	
H6	0.6272	0.6330	0.2133	0.020*	
H7	−0.0525	0.2040	0.5657	0.025*	
H8	0.1529	0.2997	0.9450	0.046*	
H9	−0.0875	0.3956	0.7543	0.032*	
H10	−0.0648	0.3742	0.8501	0.031*	
H11	0.1560	0.3806	0.6450	0.022*	
H12	0.1556	0.4022	0.7502	0.022*	
H13	−0.0783	0.2055	0.0540	0.045*	
H14	0.1057	0.2940	0.4286	0.021*	
H15	−0.1274	0.4002	0.2485	0.024*	
H16	−0.0996	0.3831	0.3450	0.024*	
H17	0.1182	0.1257	0.1545	0.030*	
H18	0.1217	0.0916	0.2420	0.030*	

### Atomic displacement parameters (Å^2^)

**Table:**

	*U*^11^	*U*^22^	*U*^33^	*U*^12^	*U*^13^	*U*^23^
Co1	0.0313 (4)	0.0051 (3)	0.0070 (3)	0.0000	0.0045 (2)	0.0000
Co2	0.0372 (4)	0.0050 (3)	0.0075 (3)	0.0000	0.0052 (2)	0.0000
S1	0.0131 (5)	0.0110 (5)	0.0113 (5)	0.0000	−0.0020 (4)	0.0000
S2	0.0133 (3)	0.0130 (4)	0.0113 (3)	0.0074 (2)	0.0034 (3)	0.0013 (2)
S3	0.0145 (5)	0.0112 (5)	0.0143 (5)	0.0000	0.0088 (4)	0.0000
S4	0.0107 (3)	0.0076 (3)	0.0075 (3)	0.0005 (2)	0.0029 (3)	−0.0007 (2)
S5	0.0114 (3)	0.0058 (3)	0.0080 (3)	0.0007 (2)	0.0011 (3)	−0.0010 (2)
O1	0.0095 (10)	0.0191 (12)	0.0240 (12)	−0.0005 (9)	0.0047 (10)	0.0014 (10)
O2	0.0136 (10)	0.0168 (12)	0.0181 (11)	−0.0003 (9)	0.0001 (9)	0.0011 (9)
O3	0.0180 (11)	0.0100 (12)	0.0089 (10)	−0.0007 (8)	0.0021 (9)	−0.0004 (8)
O4	0.0146 (10)	0.0111 (11)	0.0142 (10)	0.0021 (8)	0.0063 (9)	−0.0032 (8)
O5	0.0169 (11)	0.0101 (11)	0.0135 (10)	−0.0003 (9)	0.0009 (9)	0.0023 (9)
O6	0.0186 (11)	0.0106 (11)	0.0100 (10)	−0.0004 (9)	−0.0007 (9)	−0.0006 (8)
O7	0.0126 (10)	0.0092 (11)	0.0152 (10)	0.0031 (8)	0.0045 (9)	−0.0004 (8)
O8	0.0167 (11)	0.0077 (11)	0.0104 (10)	−0.0015 (8)	0.0001 (9)	0.0003 (8)
O9	0.042 (2)	0.0102 (17)	0.0094 (15)	0.0000	0.0008 (15)	0.0000
O10	0.095 (3)	0.0078 (18)	0.0115 (16)	0.0000	−0.015 (2)	0.0000
O11	0.0573 (18)	0.0121 (12)	0.0094 (10)	0.0091 (12)	0.0126 (12)	0.0010 (9)
O12	0.0365 (14)	0.0086 (11)	0.0108 (10)	−0.0018 (10)	0.0051 (10)	−0.0022 (9)
O13	0.094 (3)	0.0091 (18)	0.0086 (15)	0.0000	−0.009 (2)	0.0000
O14	0.034 (2)	0.0075 (16)	0.0118 (15)	0.0000	0.0012 (15)	0.0000
O15	0.0406 (15)	0.0089 (11)	0.0106 (10)	0.0045 (10)	0.0011 (11)	−0.0006 (9)
O16	0.0523 (17)	0.0089 (12)	0.0132 (11)	0.0059 (11)	0.0150 (12)	0.0015 (9)
O17	0.141 (9)	0.149 (9)	0.180 (10)	0.0000	0.054 (8)	0.0000
C1	0.0094 (13)	0.0123 (16)	0.0148 (14)	0.0035 (12)	−0.0011 (12)	0.0048 (12)
C2	0.0150 (15)	0.0087 (15)	0.0084 (13)	0.0059 (12)	0.0009 (12)	0.0008 (11)
C3	0.0148 (14)	0.0095 (16)	0.0098 (14)	0.0032 (12)	0.0016 (12)	0.0002 (11)
C4	0.0133 (14)	0.0071 (15)	0.0110 (13)	−0.0002 (11)	0.0031 (12)	0.0023 (11)
C5	0.0133 (14)	0.0090 (15)	0.0103 (14)	0.0042 (11)	0.0040 (12)	0.0007 (11)
C6	0.0132 (14)	0.0076 (14)	0.0092 (13)	0.0026 (11)	−0.0019 (12)	−0.0006 (11)
C7	0.0091 (13)	0.0132 (16)	0.0124 (14)	0.0043 (12)	0.0038 (12)	−0.0030 (12)
C8	0.0114 (14)	0.0091 (15)	0.0103 (13)	0.0006 (11)	0.0075 (12)	0.0006 (11)
C9	0.0101 (13)	0.0091 (15)	0.0088 (13)	0.0005 (11)	0.0008 (12)	−0.0016 (11)
C10	0.0128 (14)	0.0070 (15)	0.0123 (13)	0.0026 (11)	0.0031 (12)	−0.0004 (11)
C11	0.0175 (15)	0.0078 (15)	0.0077 (13)	0.0030 (12)	0.0040 (13)	−0.0013 (11)
C12	0.0129 (14)	0.0092 (15)	0.0097 (13)	0.0070 (11)	0.0027 (12)	−0.0008 (11)

### Geometric parameters (Å, °)

**Table:**

Co1—O9	2.079 (3)	C3—C4	1.392 (4)
Co1—O10	2.064 (3)	C4—C5	1.386 (4)
Co1—O11	2.085 (2)	C5—C6	1.390 (4)
Co1—O11^i^	2.085 (2)	C7—C8	1.413 (4)
Co1—O12	2.088 (2)	C7—C12	1.393 (4)
Co1—O12^i^	2.088 (2)	C8—C9	1.391 (4)
Co2—O13	2.076 (3)	C9—C10	1.394 (4)
Co2—O14	2.090 (3)	C10—C11	1.387 (4)
Co2—O15	2.078 (2)	C11—C12	1.397 (4)
Co2—O15^i^	2.078 (2)	O1—H5	0.779
Co2—O16	2.085 (2)	O2—H6	0.878
Co2—O16^i^	2.085 (2)	O9—H7	0.822
S1—C6	1.786 (3)	O9—H7^i^	0.822
S1—C6^ii^	1.786 (3)	O10—H8	0.925
S2—C2	1.786 (3)	O10—H8^i^	0.925
S2—C12	1.785 (3)	O11—H9	0.813
S3—C8	1.781 (3)	O11—H10	0.766
S3—C8^ii^	1.781 (3)	O12—H11	0.911
S4—O3	1.467 (2)	O12—H12	0.820
S4—O4	1.458 (2)	O13—H13	0.811
S4—O5	1.462 (2)	O13—H13^i^	0.811
S4—C4	1.775 (3)	O14—H14	0.776
S5—O6	1.462 (2)	O14—H14^i^	0.776
S5—O7	1.461 (2)	O15—H15	0.837
S5—O8	1.455 (2)	O15—H16	0.874
S5—C10	1.762 (3)	O16—H17	0.772
O1—C1	1.345 (3)	O16—H18	0.837
O2—C7	1.341 (3)	C3—H1	0.888
C1—C2	1.406 (4)	C5—H2	0.947
C1—C6	1.392 (4)	C9—H3	1.009
C2—C3	1.378 (4)	C11—H4	0.958
			
O9—Co1—O10	179.01 (18)	C3—C4—C5	120.3 (2)
O9—Co1—O11	89.19 (10)	C4—C5—C6	119.6 (2)
O9—Co1—O11^i^	89.19 (10)	S1—C6—C1	120.2 (2)
O9—Co1—O12	90.31 (10)	S1—C6—C5	119.3 (2)
O9—Co1—O12^i^	90.31 (10)	C1—C6—C5	120.4 (2)
O10—Co1—O11	91.51 (12)	O2—C7—C8	119.9 (2)
O10—Co1—O11^i^	91.51 (12)	O2—C7—C12	121.1 (2)
O10—Co1—O12	89.00 (12)	C8—C7—C12	119.0 (2)
O10—Co1—O12^i^	89.00 (12)	S3—C8—C7	119.9 (2)
O11—Co1—O11^i^	89.58 (11)	S3—C8—C9	119.9 (2)
O11—Co1—O12	89.31 (10)	C7—C8—C9	120.2 (2)
O11—Co1—O12^i^	178.79 (10)	C8—C9—C10	119.8 (2)
O11^i^—Co1—O12	178.79 (10)	S5—C10—C9	119.5 (2)
O11^i^—Co1—O12^i^	89.31 (10)	S5—C10—C11	119.8 (2)
O12—Co1—O12^i^	91.80 (10)	C9—C10—C11	120.5 (2)
O13—Co2—O14	179.19 (18)	C10—C11—C12	119.8 (2)
O13—Co2—O15	90.44 (12)	S2—C12—C7	120.3 (2)
O13—Co2—O15^i^	90.44 (12)	S2—C12—C11	119.1 (2)
O13—Co2—O16	90.52 (12)	C7—C12—C11	120.5 (2)
O13—Co2—O16^i^	90.52 (12)	C1—O1—H5	114.1
O14—Co2—O15	88.99 (9)	C7—O2—H6	111.3
O14—Co2—O15^i^	88.99 (9)	Co1—O9—H7	128.0
O14—Co2—O16	90.04 (10)	Co1—O9—H7^i^	128.0
O14—Co2—O16^i^	90.04 (10)	H7—O9—H7^i^	103.8
O15—Co2—O15^i^	88.64 (10)	Co1—O10—H8	130.8
O15—Co2—O16	178.36 (10)	Co1—O10—H8^i^	130.8
O15—Co2—O16^i^	90.02 (10)	H8—O10—H8^i^	98.2
O15^i^—Co2—O16	90.02 (10)	Co1—O11—H9	121.8
O15^i^—Co2—O16^i^	178.36 (10)	Co1—O11—H10	124.5
O16—Co2—O16^i^	91.31 (10)	H9—O11—H10	107.6
C6—S1—C6^ii^	99.56 (15)	Co1—O12—H11	125.2
C2—S2—C12	98.15 (14)	Co1—O12—H12	111.6
C8—S3—C8^ii^	99.04 (15)	H11—O12—H12	106.7
O3—S4—O4	111.87 (13)	Co2—O13—H13	129.0
O3—S4—O5	111.47 (12)	Co2—O13—H13^i^	129.0
O3—S4—C4	105.50 (14)	H13—O13—H13^i^	101.1
O4—S4—O5	113.74 (13)	Co2—O14—H14	123.7
O4—S4—C4	105.98 (14)	Co2—O14—H14^i^	123.7
O5—S4—C4	107.68 (13)	H14—O14—H14^i^	105.7
O6—S5—O7	111.83 (13)	Co2—O15—H15	125.6
O6—S5—O8	112.81 (13)	Co2—O15—H16	122.8
O6—S5—C10	107.21 (14)	H15—O15—H16	96.7
O7—S5—O8	113.11 (13)	Co2—O16—H17	120.4
O7—S5—C10	105.42 (14)	Co2—O16—H18	121.9
O8—S5—C10	105.79 (14)	H17—O16—H18	98.9
O1—C1—C2	119.6 (2)	C2—C3—H1	118.5
O1—C1—C6	121.1 (2)	C4—C3—H1	121.3
C2—C1—C6	119.2 (2)	C4—C5—H2	118.9
S2—C2—C1	119.7 (2)	C6—C5—H2	121.4
S2—C2—C3	120.2 (2)	C8—C9—H3	115.8
C1—C2—C3	120.0 (2)	C10—C9—H3	124.4
C2—C3—C4	120.2 (2)	C10—C11—H4	122.0
S4—C4—C3	119.1 (2)	C12—C11—H4	118.1
S4—C4—C5	120.5 (2)		

Symmetry codes: (i) *x*, −*y*+1/2, *z*; (ii) *x*, −*y*+3/2, *z*.

### Hydrogen-bond geometry (Å, °)

**Table:**

*D*—H···*A*	*D*—H	H···*A*	*D*···*A*	*D*—H···*A*
O1—H5···O1^ii^	0.78	2.17	2.877 (3)	151
O2—H6···O1	0.88	2.26	3.082 (3)	156
O2—H6···O12^iii^	0.88	2.71	3.154 (3)	113
O9—H7···O4^iv^	0.82	2.06	2.878 (3)	173
O9—H7···O4^iv^	0.82	2.06	2.878 (3)	173
O10—H8···O8^v^	0.93	1.83	2.729 (2)	163
O10—H8···O8^v^	0.93	1.83	2.729 (2)	163
O11—H9···O3^vi^	0.81	1.95	2.741 (3)	165
O11—H10···O7^vi^	0.77	2.04	2.795 (3)	169
O12—H11···O5	0.91	1.86	2.764 (3)	173
O12—H12···O6^v^	0.82	2.00	2.812 (3)	174
O13—H13···O7^vii^	0.81	1.95	2.739 (2)	165
O13—H13···O7^vii^	0.81	1.95	2.739 (2)	165
O14—H14···O5	0.78	2.05	2.818 (2)	172
O14—H14···O5	0.78	2.05	2.818 (2)	172
O15—H15···O6^viii^	0.84	1.91	2.739 (3)	170
O15—H16···O4^vi^	0.87	1.89	2.743 (3)	164
O16—H17···O8^ix^	0.77	2.01	2.764 (3)	166
O16—H18···O3^ix^	0.84	1.92	2.719 (3)	159

Symmetry codes: (ii) *x*, −*y*+3/2, *z*; (iii) −*x*+1, −*y*+1, −*z*+1; (iv) −*x*, *y*−1/2, −*z*+1; (v) *x*, *y*, *z*+1; (vi) −*x*, −*y*+1, −*z*+1; (vii) −*x*, *y*−1/2, −*z*; (viii) −*x*, −*y*+1, −*z*; (ix) *x*, −*y*+1/2, *z*.
